# Rise and rise of anesthesiologists under the shadow of COVID-19

**DOI:** 10.1186/s42077-021-00131-z

**Published:** 2021-02-03

**Authors:** Prakash Gyandev Gondode, Omshubham Asai, Abhinav Lambe

**Affiliations:** Department of Anaesthesiology, All India Institute of Medical Sciences, Nagpur, India

COVID-19 pandemic continues to overflood the emergency rooms, in-patient floors, and ICUs with patients of COVID-19, overwhelming the healthcare resources. At such challenging times of uncertainty, the world looked at anesthesiologists desperately as airway experts, respiratory physicians, and intensivists, and even asked them to assume leadership roles in an unknown and unclear scenario, apart from participating in their conventional roles as perioperative physicians.

Anesthesiologists, having a vast skill set, are extremely valuable to the COVID-19 management team, but the procedures they perform also puts them in the most danger (endotracheal intubation, open suctioning, and nebulizer treatments, which generates the highest risk for droplet exposure). Besides, emotional talks with a patient whose family is not allowed to visit, family discussions on end of care- life goals, and the realization of risk of contagion to own family members and dependants are emotionally draining and certainly takes a toll on the anaesthesiologist. The heavy workload and discomfort of wearing personal protective equipment for long durations can worsen the depression. But globally, anesthesia colleagues have reached out to each other through social media and new videoconferencing technologies to communicate, share information, and offer advice and support, which is commendable.

As COVID-19 saw an increasing number of patients requiring ventilatory support (high-flow oxygen therapy, non-invasive ventilation, invasive mechanical ventilation, and extracorporeal membrane oxygenation), there was never a dearth felt this badly of anesthesiologists. Even freshly passed anesthesia residents are coveted, which only accentuates the importance of the specialty.

This pandemic has laid its shadow on the current practices: frequently changing protocols, newer equipment, use of PPEs, induction of telemedicine in pre-anesthesia evaluation and pain clinics, use of video laryngoscope with replaceable blades, and regional over general anesthesia are the new normal (Uppal et al. 2020). Role of anesthesiology societies and organizations has also broadened: formulating protocols, testing of newer equipment, conducting informative webinars, and dispersion of information using various social media platforms and journals, along with providing psychological support for front-line anesthesiologists.

Many anesthesiologists assumed leadership roles at various institutes throughout the globe: filling roles that did not exist before COVID-19, solving problems that we have never faced before, developing clinical innovations, supporting each other, and setting up new patient care services in record times. Anesthesiologists are also working on ground zero, at various containment zones, quarantine, and isolation centers, providing basic and necessary emergency care.

The challenges thrown by the pandemic were bravely received by anesthesiologists; their hunger and interest for learning about COVID became so evident, and when the American Society of Anesthesiologists recently offered a new COVID-19 program on its website, the site temporarily crashed when an overwhelming number of members tried to access it (McCarthy 2020).

Once seldom talked about, various big media houses (The Print, The TIMES, The ABC news, etc.) are acknowledging the effort and work of an anesthesiologist during the pandemic, some even calling him a hero (Felsenthal 2020; Baldwin 2020) “Coronavirus intubation team racing against death.” This story of a team of 18 anesthesiologists and two anesthesia nurses performing nearly 50 intubations for critically ill COVID patients over 8 days has become famous. Another story that made rounds is of Dr. SL Yao, former vice-president of the Chinese Society of Anesthesiology and Wuhan Union Hospital, who was infected and hospitalized for 4 weeks in Wuhan. His participation in the international webinar from his hospital bed has inspired many (Zhang et al. 2020).

The administration must pay attention to minimize staff burnout, mental health, and boost morale while delivering quality care. High-quality training programs, Simulations, and mock drills should be a part of learning and preparedness.

From managing patients at ground zero to treating the most critical cases admitted to intensive care units (ICUs), anesthesiologists are at the forefront of the public health crisis. Hailed already for their critical role across the spectrum of patient care, and the leadership potential locally, nationally, and globally, this pandemic has indeed seen the RISE and RISE of an anesthesiologist.

## Data Availability

Not applicable.

## Abbreviations

COVID: Coronavirus disease
ICU: Intensive care unit
PPE: Personal protective equipment
